# Difficult but important questions about the ethics of qualitative research

**DOI:** 10.1007/s40037-018-0414-0

**Published:** 2018-03-13

**Authors:** Elise Paradis, Lara Varpio

**Affiliations:** 1 University of Toronto, Toronto, Canada; 2 Department of Medicine, Uniformed Services University of the Health Sciences, Bethesda, Maryland USA

We are excited to write this commentary and commend Reid and colleagues for their reflexions on their personal challenges with the ethics of qualitative research. Recently, Baker et al. [1] educated our health professions education community about the difference between procedural and practical ethics. *Procedural ethics* refers to the formal process of ethical board review, and *practical ethics* refers to the ethical decision-making that arises during research practice [1, p. 607]. These authors note the importance of authentically representing study participants, minimizing power differentials, and contributing meaningfully to knowledge. Baker et al. argue for reflexivity, which can help researchers ‘appreciate the importance of procedural ethics and respond thoughtfully to practical ethics’ (p. 607).

This is precisely what Reid and colleagues do in their piece titled ‘Ethical dilemmas and reflexivity in qualitative research’ [2]: through reflection, they make themselves and their research practices open to scrutiny. In so doing, the authors offer us glimpses into the power of reflexive research practices. Following Tracy [3], Reid et al. explore four domains of research processes and products: procedural ethics (or the ethics approval process, where the authors cover issues of integrity and altruism), situational ethics (or the research context, where they discuss access negotiations), ethical relationships between researchers and participants (where the authors explore power dynamics and role conflicts), as well as issues related to completing and disseminating findings.

Through their work, Reid and colleagues illustrate powerfully how the ethics of qualitative research involves much more than obtaining informed consent before an interview, ensuring that data are anonymized prior to analysis, or following our institutional review board’s prescriptions for how data will be stored securely. Indeed, the ethics of qualitative research are much more than “a ‘hurdle’ to be surmounted” [2]. The ethics of qualitative research live in the ‘specific and situated ethical tensions and dilemmas [that] arise during the practice of research’ [2]. Reid et al.’s piece is an important step towards making these tensions and dilemmas part of the research discourse in health professions education. Their work clearly identifies an under-explored area in health professions education research: research on practical ethics.

As qualitative researchers, we also have first-hand knowledge of the ethical challenges that are part of qualitative research. In fact, some research experiences haunted one of us (L.V.) for nearly 10 years. Only recently was L.V. able to articulate the lessons learned during a needs assessment study for global health education (GHE) as a lesson in practical ethics [4]. This 10-year delay between the experience and the sharing of that story is, at least in part, indicative of how daunting it can be for researchers to talk about ethical dilemmas. In the case of the GHE needs assessment, the research team stumbled upon stories of emotional and physical trauma experienced by learners while engaging in global health experiences. While no ethical lines were crossed during the research intervention or data collection, the interview data unveiled a problem of which the local institution was completely unaware. Close data monitoring allowed the research team to react quickly to these stories, but sharing how the situation was handled was difficult. It felt like a story that should be hidden rather than shared. It was uncomfortable. But it is those uncomfortable stories and situations that will enable all of us to be better qualitative researchers and better partners to our research participants. Instead of burying those stories, or waiting 10 years to tell them, we want to encourage readers to share their experiences of contending with the ethics of qualitative research.

Reid et al. [2] also challenge us to face the very hard question of whether research can ever be truly altruistic. As researchers, we always benefit directly from the research we conduct (through the prestige and rewards associated with grant capture and/or publishing), while our research participants rarely do: they seldom read our publications and generally are not the beneficiaries of the educational and policy changes that follow from our findings. Our community has not yet brought participatory approaches into the mainstream, but Reid et al. challenge us to consider more carefully the role of principal investigators in defining research agendas.

Finally, and unfortunately, health professions education research is still held to the ethical standards developed for clinical medicine. Qualitative research often needs to be contorted to fit ethical review forms that reflect clinical research. The kinds of phenomena that are qualitatively studied—people’s perceptions and practices—are often deemed to be without any risk to participants. Yet, as Reid et al.’s piece shows, this is simply not the case.

Qualitative researchers in our field need to take action. We must advocate for the transformation of the ethics approval process to address the ethics of qualitative research. Yes, this will likely require that we educate, and maybe even sit on, ethics review committees. We may need to get involved with the design of ethics-related educational modules that address the specificities of the ethics of qualitative research. But we contend that it is better to invest our time and energy in doing that work than wasting it on the charade that there are no ethical challenges in qualitative research.
